# Second degree AV block and severely impaired contractility in cardiac myxedema: a case report

**DOI:** 10.1186/s13044-015-0018-2

**Published:** 2015-05-19

**Authors:** Apostolos Chatzitomaris, Michael Scheeler, Michael Gotzmann, Roland Köditz, Janice Schildroth, Kathy Miriam Knyhala, Volkmar Nicolas, Christoph Heyer, Andreas Mügge, Harald H. Klein, Johannes W. Dietrich

**Affiliations:** Department of Endocrinology and Diabetes, Medical Hospital I, Bergmannsheil University Hospitals, Ruhr University of Bochum, Bochum, NRW Germany; Department of Cardiology and Nephrology, Helios Klinikum Berlin-Buch, Berlin, Germany; Department of Cardiology and Angiology, Medical Hospital II, Bergmannsheil University Hospitals, Ruhr University of Bochum, Bochum, NRW Germany; Institute of Diagnostic Radiology, Interventional Radiology and Nuclear Medicine, Bergmannsheil University Hospitals, Ruhr Univeristy of Bochum, Bochum, NRW Germany; MVZ Radiologie - Institut für Kinderradiologie, Bochum, NRW Germany

**Keywords:** Cardiac myxedema, Peri-myocarditis, Thyrocardiac axis, Hypothyroid Graves’ disease

## Abstract

The heart is a major target organ for thyroid hormone action. Severe overt hypothyroidism can result in diastolic hypertension, lowered cardiac output, impaired left ventricular contractility and diastolic relaxation, pericardial effusion and bradycardia. However, the function of the atrial pacemaker is usually normal and the degree by which the heart rate slows down is often modest. Here we report the case of a 20 year old male Caucasian with severe overt hypothyroidism. He presented with syncopation due to second degree atrioventricular block type Mobitz 2 and heart failure with reduced ejection fraction (38 %). Laboratory testing revealed a severe overt hypothyroidism with markedly elevated TSH (>100 mIU/L) and reduced fT3 and fT4 levels. The condition was caused by hypothyroid Graves’ disease (Graves’ disease with Hashimoto component). Although magnetic resonance imaging of the heart demonstrated decreased cardiac contractility and pericardial effusion, suggesting peri-myocarditis, plasma levels for BNP and troponin I were low. A possible infectious cause was unlikely, since testing for cardiotropic viruses was negative. The patient was treated with intravenous levothyroxine and after peripheral euthyroidism had been achieved, left ventricular ejection fraction returned to normal and pericardial effusion dissolved. Additionally, bradycardiac episodes abated, although intermittent second degree AV block was still occasionally present during the night. In conclusion, overt hypothyroidism may be associated by cardiac myxedema affecting both electrophysiology and contractility, observations that underscore the necessity of thyroid testing in different phenotypes of heart failure.

**Electronic supplementary material:**

The online version of this article (doi:10.1186/s13044-015-0018-2) contains supplementary material, which is available to authorized users.

## Introduction

From a clinical perspective the phenotype of hypothyroidism is modulated by patient’s age [1], the rate of its onset, and potential comorbidities.

Hypothyroidism is a known risk factor for cardiovascular disease [2]. Atherosclerosis may result from dyslipidemia [3] and diastolic hypertension [4], both conditions that may be a consequence of thyroid hormone deficiency. Myocardial dysfunction, both systolic and diastolic, is also associated with hypothyroidism; congestive heart failure may ensue, particularly in individuals with severe thyroid hormone deficiency [5]. Pericardial effusions, though usually small and of little significance, have been identified in up to 50 % of patients with thyroid gland failure [6–9]. Pericardial tamponade is rare but has been reported [10–12]. Hypothyroidism classically has been associated with bradycardia, but the degree, by which the heart rate slows down, is often modest [13, 14]. The function of the atrial pacemaker is normal and atrial ectopy is rare, however premature ventricular beats and occasionally ventricular tachycardia can occur [15]. Hypothyroidism may even be accompanied by the syndrome of torsade de pointes with a long QT interval and ventricular tachycardia, which may resolve with levothyroxine treatment [16]. Disturbances in atrioventricular conduction have been described in myxedema, but seem to be rare. The co-existence of impaired left ventricular contractility, diastolic hypertension, increased systemic vascular resistance, peripheral edema and decreased exercise tolerance suggests that hypothyroidism may result in heart failure. However, hypothyroidism as a sole cause of heart failure has been rarely reported [17–20]. Hypothyroid patients are able to increase their cardiac output and decrease systemic vascular resistance in response to exercise, unlike patients with heart failure [17, 18].

Graves’ disease is a B-cell mediated autoimmune disease induced by the expression of autoantibodies to the thyrotropin (TSH) receptor (TRAbs) as the major pathogenic feature [19–21]. There are two general types of TRAbs, which can each promote a type of autoimmune thyroiditis (AITD): TSIs (also referred to as sTRAbs) that mimic TSH stimulating the thyroid, which results in autoimmune hyperthyroidism, or classical Graves’ disease; and thyroid-blocking antibodies (TBAbs or iTRAbs), which block he binding of TSH to its receptor and can result in B cell-mediated autoimmune hypothyroidism [22–26]. Another type of hypothyroid Graves’ disease may ensue from overlap with T cell-mediated thyroiditis (Graves’ disease with Hashimoto component), where hypothyroidism ensues from destruction of thyroid tissue by cytotoxic T cells. Although overlap syndromes are common in cases of Graves’ disease they rarely lead to hypothyroidism compared with classical Hashimoto’s or Ord’s disease.

### Patient

**Fig. 1:**
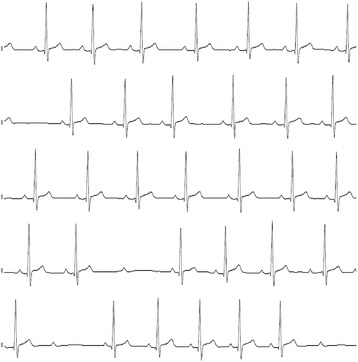
ECG monitoring in overt hypothyroid state

**Fig. 2:**
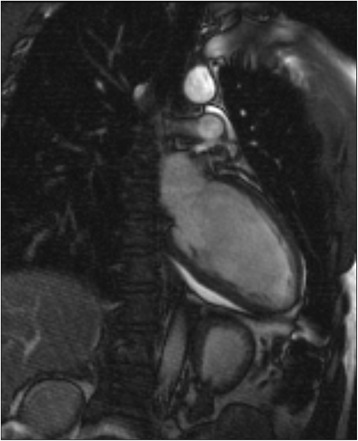
Cardiac MRT before initiation of treatment

A 20-year-old Caucasian male was admitted to the emergency department of an external secondary care hospital after he collapsed with syncopation while he watched a football match in the stadium. He had lost consciousness for less than a minute. Two years before he was diagnosed to have hypothyroidism, and the prescribed medication consisted of low dose levothyroxine and, for unknown reasons, low dose of carbimazole, potentially because of high TRAb titers. He was, however, taking his medication irregularly. Upon hospital admission he complained of increasing fatigue over the last two months. Dyspnea, chest pain, weight gain and stool irregularity were denied as well as symptoms and signs of previous viral infection in the last months. At arrival, the patient was awake and amenable, without showing any signs of alcohol consumption. His body temperature was 35.8 °C, heart rate was 52 bpm and the blood pressure was 80/40 mmHg. The physical examination was otherwise unremarkable. His initial electrocardiography (ECG) showed sinus bradycardia without atrioventricular block and ST-segment abnormalities. Echocardiography in the emergency department demonstrated impaired cardiac contractility with ejection fraction (LVEF) of 38 % and a pericardial effusion with maximal thickness of 15 mm without hemodynamic relevance. All valves were well visualized and appeared to be normal. Initial laboratory findings revealed a TSH level of over 100 mIU/L, therefore the patient was moved to our hospital in face of imminent myxedema coma. We confirmed hypothyroidism with reduced levels of plasma free thyroxine (fT4, < 3.9 pmol/L, see Table 1 for reference ranges) and plasma free triiodothyronine (fT3, 2.4 pmol/L) associated with massive rise in plasma thyrotropin (TSH, 108 mIU/L). Creatin kinase (CK) was 437 U/L (normal < 172) and CK-MB was 34 U/l (normal < 24). However, troponin I and BNP concentrations were low. Serum electrolytes, complete blood count, and basal levels of cortisol and ACTH were normal. The patient was transferred to the ICU, where treatment with intravenous infusion of levothyroxine (500 μg over the first 24 h) was commenced. ECG monitoring revealed an intermittent atrioventricular block type Mobitz 2 (Fig. 1). In order to evaluate the pericardial effusion and the impaired left ventricular ejection fraction, magnetic resonance imaging was performed, which confirmed the echocardiographic findings. A potential peri-myocarditis was susptected because of the increased signal intensity in the epicardial layer, suggesting local edema, in the T2-weighted and late gadolinium enhancement images (Fig. 2 and Additional file 1). Therefore, to rule out a peri-myocarditis, serologic studies, including parvovirus-B19, streptococcus, influenza-virus, mycoplasma, adenovirus, enterovirus, herpes virus type 6 and cytomegalovirus were performed, which did not support the diagnosis.

**Table 1:** Progression of thyroid hormone levels, ejection fraction and cardiac biomarkers after substitution therapy with levothyroxine had been initiated. Reference ranges are reported with units of measurement

Days after admission	0	0	1	2	5	9	28	83	120
	(external)								
*Concentrations of thyroid hormones*									
TSH	>100	108.34	>100	>100	>100	78.65	30.31	13.86	7.61
(0.35–3.5 mIU/L)									
Free T3		2.4	2.7	2.7	3.1	4.5	4.2	4.2	4.4
(3.5–6.3 pmol/L)									
Free T4		<3.9	<3.9	<3.9	6.44	9.0	12.87	12.87	16.73
(8–18 pmol/L)									
*Echocardiography and MRT*									
EF	25		38			60		55	58
(55–70 %)									
Cardiac output					3.2			6.0	
(2.8–8.8 L/min)									
*Concentrations of cardiac biomarkers*									
CK		437	311	223	154				
(0–172 U/L)									
CK-MB		34	24						
(<24 U/l)									
Troponin I		<0.04							
(0–0,04 μg/l)

**Fig. 3:**
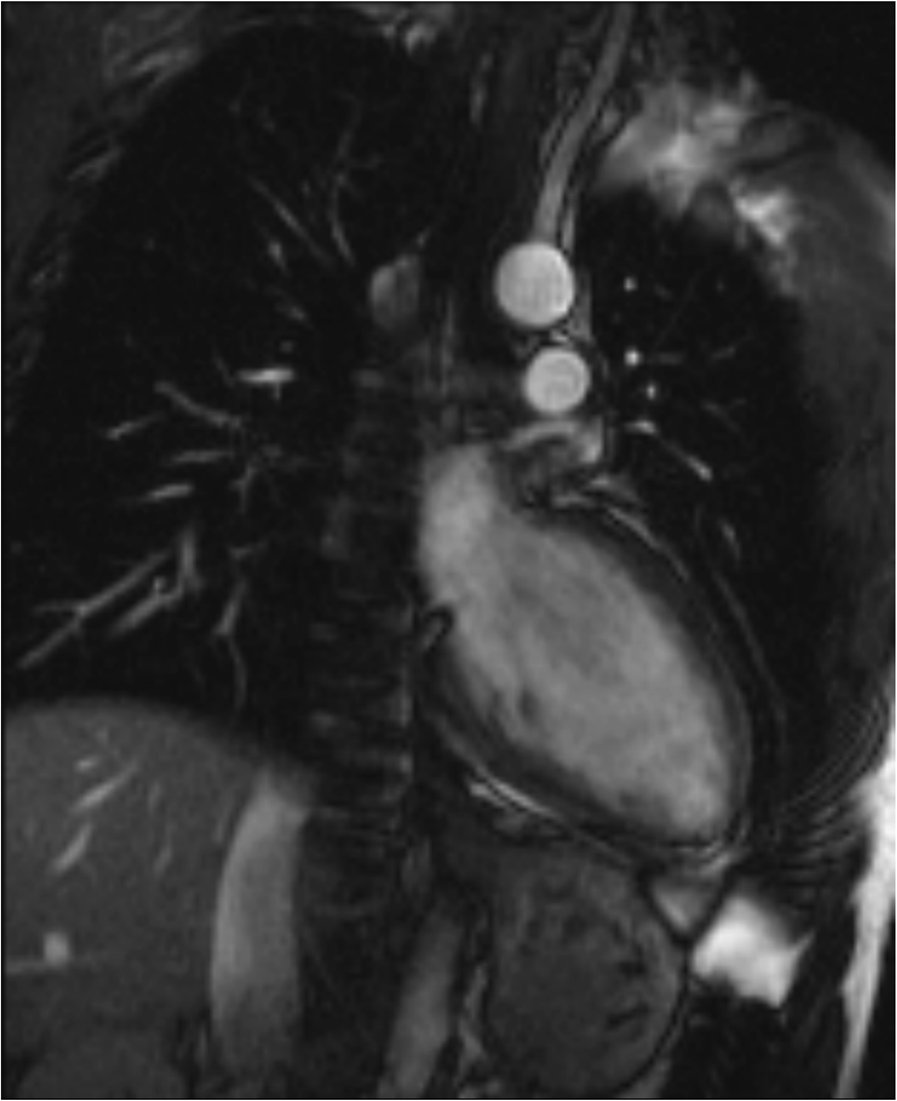
Cardiac MRT in peripheral euthyroid state

Titres of three subtypes of anti-thyroid antibodies were elevated, suggesting hypothyroid Graves’ disease with Hashimoto component (TgAb > 2000 U/mL, TPO-Ab > 3000 U/mL, TRAb 5,9 U/mL). After 3 days the intravenous levothyroxine therapy was terminated and oral substitution continued with a daily dose of 150 μg for 7 days, followed by 125 μg daily. After 7 days an echocardiography revealed normal LVEF and the pericardial effusion had vanished. When peripheral euthyroidism was achieved after 9 days, 24-h Holter ECG monitoring demonstrated regression of the bradycardiac episodes, however an intermittent atrioventricular block Mobitz type 2 during the night remained. A pacemaker was not considered because of a rise in the cardiac frequency during training. In a follow-up MRI investigation after 3 weeks, the initially increased signal intensity in the epicardial layer in T2-weighted and late gadolinium enhancement images had been dissolved (Fig. 3 and Additional file 2). Cardiac output had improved to 6.0 L/min from formerly 3.2 L/min (Table 1).

## Discussion

Pericardial effusion and impaired cardiac contractility are common signs in hypothyroidism, and several cases of cardiac tamponade due to hypothyroidism have been described in the literature [6–12, 27–29]. Both abnormalities have been described to be reversible after achievement of a euthyroid state. In our patient both cardiac contractility and the pericardial effusion normalized or dissolved after 9 days of treatment after euthyroidism was reached. Disturbances in the conduction system of the heart are infrequent in hypothyroidism [30, 31] and, conversely, hypothyroidism is a rare cause of advanced atrioventricular block in young individuals [32]. Affected patients with second and/or third degree AV block in the setting of thyroid dysfunction almost always have to be provided with a permanent pacemaker even after thyroid status has been normalized [13, 14, 33–36]. The reason for this is unclear. However, on a molecular level the clinical phenomena found in hypothyroid-associated cardiac disease may be explained by pleiotropic effects of thyroid hormones on both gene expression and metabolism of myocardial cells. Genomic mechanisms involve T3 binding to TRs, which regulate transcription of specific cardiac genes. Positively regulated are alpha-myosin heavy chain, sarcoplasmic reticulum Ca^2+^-ATPase, Na^+^/K^+^-ATPase, beta1-adrenergic receptor, atrial natriuretic hormone and voltage-gated potassium channels (Kv1.5, Kv4.2, Kv4.3). Negatively regulated are beta-myosin heavy chain, phospholamban, adenylyl cyclase catalytic subunits, thyroid hormone receptor alpha1 and Na^+^/Ca2^+^ exchanger. Non-genomic mechanisms include direct modulation of membrane ion channels. Additional extracardiac effects of thyroid hormones on cardiovascular hemodynamics include stimulation of tissue thermogenesis, increase of cardiac output and pulmonary pressure, and decrease of both systemic vascular resistance and diastolic blood pressure with consecutively reduced afterload. Isovolumic relaxation time, as a measure of diastolic function, is increased in subclinical and even more in overt hypothyroidism. Cardiovascular risks associated with hypothyroidism include impaired cardiac contractility and diastolic function, increased systemic vascular resistance, possibly caused by decreased endothelial-derived relaxation factor, and elevated levels of serum cholesterol, C-reactive protein and homocysteine [37]. Abnormal cardiac bioenergetics in hypothyroidism is improved by substitution with levothyroxine [38].

In addition, regulation of beta-receptor density by thyroid hormones, as described on both genomic and non-genomic levels, could play a key role in this scenario [39, 40]. Fast non-genomic effects may be mediated by microtubule assembly [41].

Moreover, thyroid hormones have profound effects on myocardial calcium and potassium channels on both RNA and protein level. Hypothyroidism-induced remodelling of K^+^ channels expression is assumed to be responsible for the blunted response of peak transient outward current (Ito) and sustained current (Isus, a mix of IK, slow and Iss) to changes in membrane potential [42].

Undiagnosed and/or untreated hypothyroidism may pose a threat of sudden cardiac death, which may be mirrored by myocardial ventricular late potentials in overt hypothyroidism [43].

## Conclusion

Hypothyroidism is a differential diagnosis of suspected peri-myocarditis. This case underscores the clinical relevance of thyrocardiac interaction. Evaluation of thyroid homeostasis should be performed early in the work-up of heart failure and every kind of arrhythmia. Even though electrophysiological scars may outlast myxedema, it may be life-saving to commence substitution therapy with thyroid hormones timely.

### Consent

Written informed consent was obtained from the patient for the publication of this report and any accompanying images.

## Additional files

Additional file1:
**Sequence of cardiac MRT before initiation of treatment.**
Additional file 2:
**Sequence of cardiac MRT in peripheral euthyroid state.**
